# Isolated Gallbladder Tuberculosis Mimicking Chronic Cholecystitis: A Case Report

**DOI:** 10.1155/crhe/9990210

**Published:** 2026-01-27

**Authors:** Ananya Vig, Dikshit Chawla, Parth Dhamija, Ashwani Kumar, Mansi Singh

**Affiliations:** ^1^ MS3, Government Medical College, Patiala, Punjab, India, calicutmedicalcollege.ac.in; ^2^ MS4, Government Medical College, Patiala, Punjab, India, calicutmedicalcollege.ac.in; ^3^ Department of Surgery, Government Medical College, Patiala, Punjab, India, calicutmedicalcollege.ac.in; ^4^ Department of Medicine, Bogomolets National Medical University, Kyiv, Ukraine, nmu.edu.ua

**Keywords:** abdominal tuberculosis, case report, cholelithiasis, chronic cholecystitis, extrapulmonary tuberculosis, gallbladder tuberculosis, granulomatous inflammation, histopathological diagnosis

## Abstract

Isolated gallbladder tuberculosis (GBTB) is a rare disease, even in endemic areas, and is often misdiagnosed due to its nonspecific clinical and imaging findings. Histopathological evaluation, demonstrating granulomatous inflammation with caseous necrosis, remains the only definitive method of diagnosis. Thus, maintaining a high index of suspicion and routine histological assessment of resected specimens are vital for its timely management, especially in patients with a history of tuberculosis. We report a similar case in a 50‐year‐old male patient with imaging suggestive of gallbladder perforation and chronic cholecystitis, which later revealed a cryptic GBTB dug up during histopathological examination.

## 1. Introduction

Gallbladder tuberculosis (GBTB) is a rare infectious disease, constituting only 1% of abdominal tuberculosis cases [1]. It is uncommon even in areas of endemicity due to the gallbladder’s high resistance to tubercular infections [2]. With less than 180 cases of GBTB reported in the literature, its diagnosis remains challenging even for experienced clinicians [3]. The nonspecific pathognomonic presentation and lack of preoperative diagnostic tests often lead to misdiagnosis as chronic cholecystitis or gallbladder malignancy [4–6].

We report the case of a 50‐year‐old patient who presented in the OPD with a chief complaint of pain in the right upper abdomen, initially diagnosed with chronic cholecystitis, for which he underwent laparoscopic cholecystectomy under general anesthesia. However, he was later diagnosed with GBTB on histopathological examination. This manuscript aims to increase awareness and suspicion in identifying the rare cases of GBTB, which will be essential in eradicating tuberculosis from society.

## 2. Case Presentation

A 50‐year‐old male presented to the outpatient department with chief complaints of recurrent pain in the right upper abdomen for the past 4 months, along with dyspepsia, weight loss, and loss of appetite. No history of fever, night sweats, nausea, vomiting, jaundice, or cough with expectoration was reported. The patient had been diagnosed with gallbladder perforation 3 months before admission based on ultrasound and contrast‐enhanced computed tomography (CECT) whole abdomen findings, managed conservatively with antibiotics and analgesics, and advised to undergo cholecystectomy within the next 2 months. However, he deferred surgery and returned with a recurrence of more severe symptoms and additional complaints. He had a history of pulmonary tuberculosis 30 years back, for which he took anti‐tubercular therapy (ATT) for 6 months as per the National Tuberculosis Elimination Programme (NTEP).

The general physical and abdominal examinations were unremarkable. Routine laboratory investigations showed slightly elevated serum bilirubin (2.4 mg%) and serum glutamic‐oxaloacetic transaminase (SGOT) (54 U/L) levels. Ultrasound of the abdomen (Figure 1) revealed a thickened, edematous gallbladder (7.5 mm) with a narrow lumen and a few hypodense, echogenic masses with positive DAS suggestive of cholelithiasis. Magnetic resonance cholangiopancreatography (MRCP) images showed a contracted gallbladder with few intraluminal voids in the neck (largest measuring 17 mm), diffuse wall thickening (up to 10 mm), and a streak of fluid in the pericholecystic region, causing adhesions to the liver, duodenum, and hepatic flexure. A diagnosis of chronic cholecystitis with concealed gallbladder perforation was considered, and the patient was scheduled for laparoscopic cholecystectomy.

**Figure FIGURE 1:**
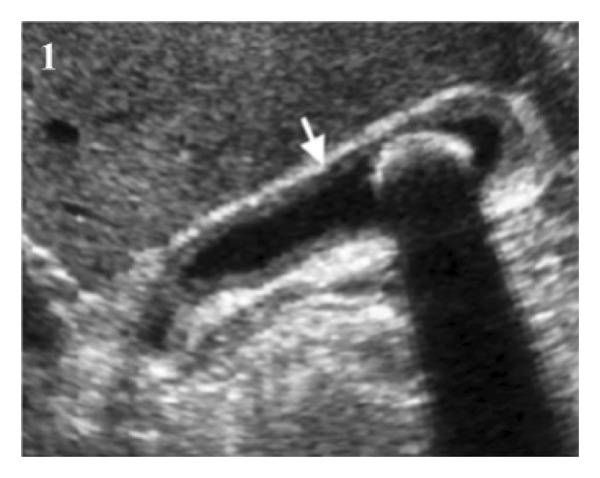
Ultrasound showing diffuse gallbladder wall thickening with stones.

Intraoperatively, a thickened, adherent, and distended gallbladder was visualized and resected from the liver bed. The cut section of the specimen showed an obliterated lumen and numerous chalky white areas extending into the peritoneal layer. Histopathological examination (Figure 2) revealed chronic granulomatous inflammation with caseous necrosis, multinucleated giant cells, and Langhans giant cells, confirming GBTB. The patient’s postoperative recovery was uneventful. The patient was prescribed ATT as per the guidelines of the NTEP of India with isoniazid (I), rifampicin (R), ethambutol (E), and pyrazinamide (P) for 6 months and was scheduled for a follow‐up after completing the full course. The patient was followed for 6 months postoperatively and was further lost to follow‐up for long‐term outcomes.

**Figure FIGURE 2:**
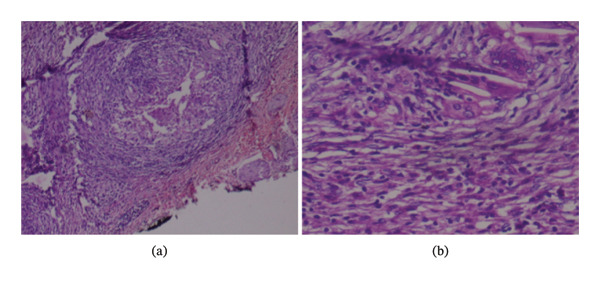
Histopathology of the resected gallbladder specimen: low‐power view of gallbladder showing necrotizing granulomas (a) composed of epithelioid cells and Langhans type multinucleated giant cells (b).

## 3. Discussion

GBTB remains one of the rarest manifestations of extrapulmonary tuberculosis, accounting for approximately 1% of abdominal TB cases [2, 7]. The gallbladder’s inherent resistance, attributable to the bactericidal properties of bile, renders it an uncommon site for *Mycobacterium tuberculosis* infection. This natural defense leads to a low index of preoperative clinical suspicion, frequently leading clinicians to overlook tuberculosis as a potential etiology in gallbladder pathology.

In the present case, the clinical and radiological features closely mimicked more common conditions such as chronic cholecystitis and gallbladder perforation [6, 8]. The imaging studies revealed findings that were not pathognomonic and could easily be confused with cholelithiasis or even gallbladder malignancy. Such overlap in presentation complicates the differential diagnosis, particularly in regions where gallbladder carcinoma and benign inflammatory conditions are more routinely encountered.

A notable aspect of our patient’s history is a remote episode of pulmonary tuberculosis, which should prompt a heightened clinical suspicion for extrapulmonary reactivation, even decades after the initial infection [2, 7]. Although antecedent TB may seem clinically insignificant over time, it serves as a crucial diagnostic clue in cases with atypical presentations. This underlines the imperative that every cholecystectomy specimen be rigorously examined histopathologically to detect granulomatous inflammation and caseous necrosis, the hallmarks of tubercular infection.

## 4. Limitations

This case report describes a single patient with GBTB, and therefore the findings cannot be generalized to all patients with gallbladder disease or extrapulmonary TB. The disease was not considered preoperatively in the differential diagnosis because the patient did not present with signs or symptoms of pulmonary or extrapulmonary TB (no H/o cough, fever, night sweats, and no palpable lymph nodes); consequently no preoperative TB‐specific diagnostic tests were undertaken before scheduling cholecystectomy. As the patient was lost to follow‐up, contact tracing, screening of contacts, and further evaluation could not be done.

## 5. Conclusion

This case report contributes significantly to the current literature by highlighting the diagnostic challenges associated with GBTB and reinforcing the importance of incorporating a thorough patient history and mandatory histopathological evaluation in managing gallbladder diseases. By advocating for a meticulous approach in patients with previous TB exposure and ambiguous clinical presentations, our findings aim to improve diagnostic accuracy, incorporate stringent protocols for such cases, and optimize patient outcomes in regions where tuberculosis remains endemic [6, 8, 9].

## Funding

This research received no specific grant from funding agencies in the public, commercial, or not‐for‐profit sectors.

## Conflicts of Interest

The authors declare no conflicts of interest.

## Data Availability

All data generated or analyzed during this study are available from the corresponding author upon reasonable request.
